# Super-High Magnification Dermoscopy in 190 Clinically Atypical Pigmented Lesions

**DOI:** 10.3390/diagnostics13132238

**Published:** 2023-06-30

**Authors:** Elisa Cinotti, Vittoria Cioppa, Linda Tognetti, Jean Luc Perrot, Renato Rossi, Matteo Gnone, Alessandra Cartocci, Pietro Rubegni, Giulio Cortonesi

**Affiliations:** 1 Department of Medical, Surgical and Neurological Science, Dermatology Section, University of Siena, S. Maria alle Scotte Hospital, 53100 Siena, Italy; 2 Department of Dermatology, University Hospital of Saint-Etienne, 42055 Saint-Etienne, France; 3 Department of Dermatology, Skin Center Senigallia, 60019 Ancona, Italy; 4 Dermatology Private Practice, 16121 Genoa, Italy; 5 Department of Medical Biotechnology, University of Siena, 53100 Siena, Italy

**Keywords:** dermoscopy, high magnification, super-high magnification, keratinocyte, melanocyte, melanoma

## Abstract

Super-high (×400) magnification dermoscopy (D400) is a new non-invasive imaging technique that has been shown to add information for the differential diagnosis of melanocytic lesions in a pilot study. Our study aimed to confirm if D400 can add details for the discrimination of clinically atypical nevus and melanoma. This is a retrospective observational, multicentric study enrolling patients who received ×20 (D20) and ×400 (D400) magnification dermoscopic examinations of pigmented skin lesions. Dermoscopic images were retrospectively evaluated by three observers for the presence/absence of nine D20 and twenty D400 dermoscopic features defined in the previous pilot study. Univariate and multivariate analyses were carried out to find predictors of benign and malignant behaviors. At D20, an atypical pigment network, blue-whitish veil, atypical vascular pattern, regression, and homogenous pattern were more frequent in melanoma than in nevi (*p* < 0.001). At D400, melanoma showed more frequently than benign lesions, melanocytes with an irregular arrangement and irregular in shape and size (*p* < 0.001). A network with edged papillae was more frequent in benign lesions than melanomas (*p* < 0.001). Our study confirms that D400 can identify melanocytes with an irregular arrangement, and irregularities in shape and size in melanomas, adding information to the conventional D20 examination.

## 1. Introduction

Melanoma (MM) is a malignant tumor that arises from melanocytes and primarily involves the skin, but can also arise in the eye, meninges and on various mucosal surfaces [1]. Cutaneous melanoma represents a public health issue, due to its high morbidity and mortality. [2,3,4]. The incidence and mortality rates of cutaneous melanoma differ widely by country [5]. The incidence of melanoma is increasing worldwide in particular among white populations, especially where fair-skinned people have excessive sun exposure. Earlier diagnosis of melanoma (MM) improves the survival rates but the clinical presentation can be subtle and varied [6].

Four main different subtypes of melanomas can be identified clinically and histologically: superficial spreading MM, nodular MM, lentigo maligna MM and acral lentiginous MM. In addition to these main types, there are several rarer variants of melanoma, such as desmoplastic, amelanotic and polypoid melanomas, which constitute less than 5% of cases [1].

Clinically atypical nevi span a continuum from minimally abnormal nevi to markedly abnormal nevi that clinically cannot be distinguished from MM [7]. For this reason, diagnosis of MM is based on histopathology, with subsequent treatment decisions being based on histological classification and risk calculation [8].

In addition to visual evaluation, supportive imaging techniques have been shown to improve clinical diagnostic accuracy. The most routinely used non-invasive imaging technique for the skin is dermoscopy, also known as epiluminescence microscopy, a magnifying handheld optical device that uses a light source to inspect skin lesions unobscured by skin surface reflections. Use of dermoscopy requires training, but when appropriately used, this method substantially enhances the diagnosis of unclear or doubtful lesions that are suspected to be MM, reducing the number of skin lesions that should be excised to find a MM [9]. With dermoscopy, the MM detection proportion can be increased to up to 90%, instead of 70% with only clinical inspection [10]. In addition, other non-invasive imaging procedures such as reflectance confocal microscopy (RCM) and fluorescence advanced dermoscopy can be used to improve the clinical diagnostic accuracy [11,12].

Recently, a pilot study has shown that ×400 dermoscopy (D400 or super-high magnification dermoscopy) can also aid the non-invasive diagnosis of MM by observing single pigmented cells [13,14]. Compared with nevi, MMs had a higher frequency of scattered, large, irregular (in shape and size) dendritic/roundish cells corresponding to atypical melanocytes, and violet/blue pigmented cells corresponding to melanophages under D400. The current study aimed to confirm if D400 can add details for the discrimination of clinically atypical melanocytic lesions.

## 2. Materials and Methods

### 2.1. Study Design

Retrospective observational, multicentric study.

### 2.2. Setting

Data were collected on patients who came to the Dermatology Departments of the University Hospitals of Siena (Italy) and Saint-Etienne (France), to the Department of Dermatology of the Skin Center of Senigallia (Italy), and to a Dermatology private practice in Genoa (Italy) for a dermatological examination between the 1 January 2018 and 31 December 2020. Data were analyzed from March 2022 to April 2022. The study was conducted according to the criteria set by the Declaration of Helsinki. All data were de-identified before use. The patients represented in this manuscript gave written informed consent to publication of their case details.

### 2.3. Participants

We enrolled non-consecutive patients with pigmented skin lesions of the body (except the face, palm and sole) that needed to be removed or followed up for their atypical clinical and/or ×20 dermoscopy (D20) features according to a skin imaging expert dermatologist (E.C., J.L.P., R.R., M.G.).

### 2.4. Data Sources

For this study, we only selected patients who received a D20 and D400 magnification dermoscopic examination of lesions that needed follow-up or excision due to their atypical dermoscopic features. These lesions were recorded with the videodermoscope Fotofinder Medicam 1000 (Fotofinder System, Bad Birnbach, Germany).

To perform D400, we used the same camera as D20 and we changed the terminal lens. Specialists in skin imaging (E.C. and J.L.P.) acquired at least seven images for each lesion, for a total of 1400 images of 190 skin lesions, as D400 does not show an entire lesion (D400 field of view -FOV- of 1 mm × 0.5625 mm). We included cases with histological diagnosis or lesions unmodified at clinical and dermoscopic follow-up of at least 12 months.

### 2.5. Variables

For the clinical variables, we evaluated the patients’ sex and age. A group of three dermatologists (E.C. with >10 years of experience in skin imaging, G.C. with 5 years of experience in skin imaging, and V.C. with 2 years of experience in skin imaging) belonging to the University Department of Dermatology of the University Hospital of Siena together evaluated the images.

The D20 dermoscopy variables included: general dermoscopic pattern (homogenous, globular, network) and 7-point checklist parameters (atypical pigment network, blue-white veil, atypical vascular pattern, irregular streaks, regression structures, blotches irregularly distributed, and irregular dots/globules).

The D400 variables were those described in the previous pilot study [7,8]: the presence of the pigmented cells and their features, out-of-focus blue or grey/brown structureless areas, vessels, angled nests, and networks with or without edged papillae. Pigmented cells were differentiated into keratinocytes (seen as regular polygonal brown mostly in-focus cells, evenly spread and/or inside a network), roundish melanocytes (seen as large roundish brown-to-violet/blue scattered cells—cells were defined as “large” when they were larger than keratinocytes), dendritic melanocytes (dendritic brown-to-violet/blue scattered cells), and melanophages (large blue-to-violet non-in-focus cells with a not defined polymorphous shape). Considered cell features were cell color (violet and blue colors are difficult to differentiate with D400 and were considered together, while light and dark brown were also considered together because brown is often present with multiple shades in the same structure), shape and size irregularity of melanocytes, and irregular arrangement of single melanocytes.

### 2.6. Statistical Analysis

Descriptive statistics were performed: absolute frequencies and percentages were calculated for qualitative variables and mean and standard deviations for the quantitative ones. The association between qualitative variables and the outcome (i.e., MM/nevus) and D20 or D400 was evaluated by Fisher exact test. *T*-test was carried out if the variables were normally distributed (normal distribution evaluated by Kolmogorov–Smirnov test) and there was homoscedasticity between variances evaluated by Bartlett test; otherwise, the Mann–Whitney test was used. Chi-squared test was performed to evaluate the association of D400 with anatomic site, within melanoma and nevi. The multiple Fisher exact test with Bonferroni correction was used as post hoc analysis. Logistic regression was later performed to evaluate variables that were statistically significant in the previous univariate analysis (*p*-value < 0.05). The best subset of variables was selected by a stepwise procedure based on Akaike’s criterion. Odds ratio (OR) and 95% confidence intervals (CI) were estimated by logistic regression. The analyses were carried out by R software version 3.6.2.

## 3. Results

### 3.1. Participants and Lesion Data

In this study 190 patients, comprising 91 (48%) women and 99 (52%) men (range 9–97 years) with a single atypical skin lesion of the body (except the face), were selected. The 190 skin lesions included 73 MMs and 117 benign lesions (including 17 Reed-Spitz nevi) (Table 1).

A statistically significant (*p* < 0.001) correlation between the presence of benign lesions in young patients and melanomas in elderly patients has been demonstrated (48.79 (19.19%) vs. 64.98 (16.70%)).

There was no association between sex and benign or malignant skin lesions.

### 3.2. ×20 Dermoscopy

The D20 data are reported in Table 2.

**Figure 1:**
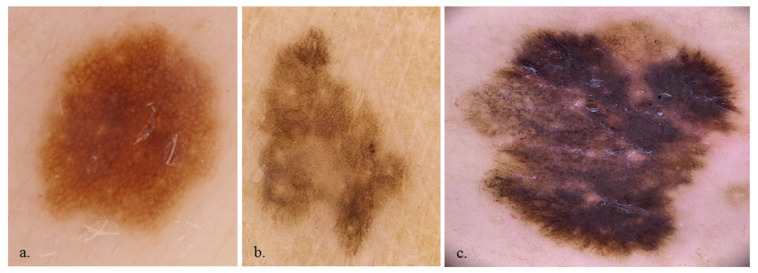
×20 dermoscopy evaluation of a nevus with a regular dermoscopic aspect (**a**), a dermoscopically atypical nevus (**b**) and a melanoma (**c**).

Concerning malignant benchmarks, the atypical pigment network (in 59 MMs (80.8%) and 57 nevi (48.7%) *p* < 0.001, Table 1), blue-whitish veil (in 49 MMs (67.1%) and 18 nevi (15.4%) *p* < 0.001, Table 2) atypical vascular pattern (in 12 MMs (16.4%) and 1 nevus (0.9%) *p* < 0.001, Table 2), irregular streaks (in 22 (30.1%) MMs and 11 (9.4%) nevi, *p* = 0.001), and regression structures (in 49 MMs (67.1%) and 33 (28.2%) *p* < 0.001, Table 2) were more frequent in MM than in the benign lesions. As for dermoscopy general patterns, the homogenous pattern was more frequent in MM (43 MMs (58.9%) and 32 nevi (27.4%); *p* < 0.001, Table 2) than in the other lesions, whereas the globular pattern was more frequent in benign lesions (in 7 MMs (9.6%) and 41 nevi (35.0%); *p* < 0.001, Table 2) (Figure 1).

### 3.3. ×400 Dermoscopy

The D400 features data are reported in Table 3.

**Figure 2:**
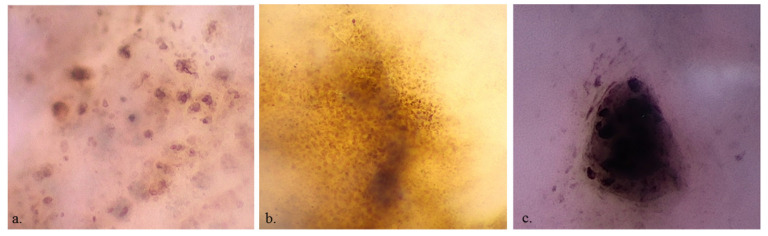
×400 dermoscopy shows pleomorphic roundish and dendritic melanocytes (**a**), homogeneous keratinocytes (**b**) and a cluster of melanocytes (**c**).

**Figure 3:**
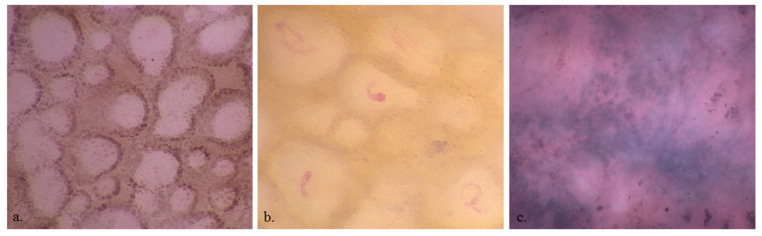
×400 dermoscopy shows edged papillae (**a**), vessels (**b**) and out-of-focus structureless areas (**c**).

MMs more frequently showed roundish (36 (49.3%) vs. 35 (29.9%); *p* = 0.011), dendritic (22 (30.1%) vs. 19 (16.2%); *p* = 0.037), irregularly arranged (30 (41.1%) vs. 19 (16.2%); *p* < 0.001), and irregular in shape and size melanocytes (38 (52.1%) vs. 21 (17.9%); *p* < 0.001), and angled nests (16 (22.2%) vs. 9 (7.7%); *p* = 0.008) than nevi (Figure 2). Benign lesions more frequently revealed a network with edged papillae than MMs (30 (25.6%) vs. 3 (4.1%); *p* < 0.001) (Figure 3). Cell color (black 13 (11.1%) vs. 7 (9.6%), brown 107 (91.5%) vs. 66 (90.4%), violet/blue 45 (38.5%) vs. 29 (39.7%)), out-of-focus bluish (36 (30.8%) vs. 24 (32.9%))or grey/brown (23 (19.7) vs. 11 (15.1%)) structureless areas and vessel presence (26 (22.2%) vs. 23 (31.5%)) were not statistically significant for the differential diagnosis between MM and benign lesions. The observation of benign lesions showed a higher visibility of keratinocytes (109 (93.2%) vs. 64 (87.7%)) and roundish nests (42 (35.9%) vs. 22 (30.1%)) and a lower presence of melanophages [(19 (16.2%) vs. 17 (23.3%))] with no statistically significant difference.

### 3.4. Multivariate Analysis

The multivariate regression considered the following variables: clinical features (age, sex and location), D20 observations (7-point checklist, general dermoscopic pattern) and D400 observations (cell presence, cell color, melanocyte irregularity in shape and size, irregular melanocyte arrangement, roundish nests, out-of-focus structureless areas, vessels, network with edged papillae and angled nests) (Table 4).

According to the stepwise procedure, older age and irregular melanocyte arrangement at D400 were more indicative of malignant lesions (OR 1.05, 95% CI 1.03–1.09, *p* < 0.001 and OR 4.69, 95% CI 1.58–15.45, *p* < 0.001, respectively), and a network with edged papillae at D400 was more indicative of benign lesion (OR 0.16, 95% CI 0.03–0.57, *p* = 0.009).

Few differences were found for 400× features based on body localization with stratification for nevus and melanoma. In melanomas, roundish melanocytes were more visible on the trunk versus the upper limbs (41.7% vs. 33.3%, *p* < 0.002, respectively) and on the lower limbs versus the upper limbs (70.6% vs. 33.3%, *p* < 0.002, respectively). As for nevi, out-of-focus grey/brown structureless areas were more present on lower limbs compared with upper limbs (38.9% vs. 8.0%, *p* < 0.035, respectively). All these date are collected in the Supplementary Materials.

## 4. Discussion

Conventional dermoscopy provides images that are at 10–30× magnification. Since its development there has also been an interest in exploring higher magnifications. Higher magnification has mainly been applied to a better identification of parasites like *Sarcoptes scabies*, that is seen as a barely visible dark triangle in conventional dermoscopy and as a well-defined oval body with possible near droppings and eggs at 70× [15].

In 1993, ×400 images of melanocytic lesions were published, but their resolution was low, and their interpretation remained vague [16]. Lower magnification at 10–30× had the advantage of giving an image of an entire skin tumor in most of the cases. This is probably the reason for the selection of this magnification in clinical practice, with terminology, diagnostic algorithms and scores that have been based on this magnification. In 2018, the Italian group of Renato Rossi published amazing images of nevi that compared ×20 and ×400 dermoscopy, highlighting the potentiality of high magnification [17]. In the following year, Jean Luc Perrot had the patience to explore entire skin lesions with D400 and handheld RCM to find the exact same area under both techniques [13]. Professor Perrot showed the same pigmented keratinocytes and pigmented atypical melanocytes with both techniques in a solar lentigo and a MM, respectively. This was the first demonstration of the possibility of observing single cells with a dermoscope device.

Subsequently, D400 also showed the possibility of observing filaments and conidia in a case of tinea nigra, highlighting how high magnification could have an impact on the identification of specific pigmented structures [18]. In addition, our group demonstrated that even non-pigmented structures could be observed under D400, such as demodex. *Demodex folliculorum* can be observed as an elongated body similar to what can be seen by RCM due to the similar size of the field of view (FOV) of these two imaging techniques [19].

There are few videodermoscopes that provide super high magnification images and the most studied are the Fotofinder described in the present work (FOV 1 mm × 0.5625 mm) and the Horus device (Horus system, Trapani, Italy, FOV 1.7 mm × 1.3 mm). Their magnification has been called super high magnification to distinguish it from high magnification (70–100×) already used in the past. The Horus device has also the possibility of producing fluorescence dermoscopy images using a monochromatic light emitting source with a wavelength of 405 nm (±5 nm) and a fixed angle of incidence [11,20]. The latter images are in a greyscale and with a FOV of 340 µm.

Our study confirmed that D400 can identify single pigmented cells in the skin, being able to reveal them in most cases. Three main pigmented cell populations seem to be identifiable under D400: keratinocytes, melanocytes and melanophages. Keratinocytes are seen as small and polygonal cells, whereas melanocytes can be identified as dendritic cells or as roundish cells larger than keratinocytes. As with RCM, it seems that is not possible to identify non-activated melanocytes that have the same size as the surrounding keratinocytes. This probably explains why melanocytes were more frequently seen in MMs than nevi.

Interestingly, we confirmed that melanocytes with the irregular arrangement (scattered) and irregular in shape and size were present at a higher frequency in MMs than in nevi (Table 3, *p* < 0.001). Cell distribution seems to be a fundamental parameter for the detection of MM at D400 because in nevi we could see homogeneously distributed keratinocytes or melanocytes organized in nests, whereas in MMs we had a higher frequency of scattered melanocytes. Based on our experience (data unpublished), these scattered cells correlate with pagetoid cells in the upper layers of the epidermis under RCM and are usually more abundant than in RCM images because they correspond to cells contained in a superposition of layers and not to a single focal plane.

The presence of edged papillae was associated with benignity, similar to what is seen under RCM, due to the lack of atypical melanocyte proliferation that alters the dermo-epidermal junction. Unlike in the previous pilot study [9], blue out-of-focus structureless areas, violet/blue pigmented cells, and melanophages were not associated with MMs, and therefore these features seem to be related to other conditions such as regression rather than malignancy.

The main limitation of D400 seems to be the poor image penetration in case of hyperkeratotic or heavily pigmented lesions or clinically bluish lesions; in these cases, it is possible to recognize only scales or heavily pigmented keratinocytes that mask the underlying structures or a bluish homogeneous color with no visible cells. If we compare D400 to RCM, D400 gives less false-positive results caused by the presence of dendritic Langerhans cells in the epidermis mistaken for melanocytes under RCM and it has a lower cost. However, D400 can miss atypical melanocytes that are not heavily pigmented or are located more deeply and can show large cells suggestive of atypical melanocytes when multiple keratinocytes are superposed due to the lack of confocal sections.

Notably, nevi of our series were clinically and dermoscopically atypical, as demonstrated by the high percentage of cases with a 7-point checklist score ≥3 (49/117 benign lesions—41.9%). As expected, some parameters of the 7-point checklist (atypical pigment network, blue-whitish veil, atypical vascular pattern, and regression structures) were more frequently linked to MM diagnosis (70/73 malignant lesions—95.9%) (*p* < 0.001) [10]. It is possible that if we consider less atypical nevi, the differences between nevi and MMs would be even more marked at D400.

The main limitation of this study was that the acquisition of the images was dependent on the expertise of the investigators. Unlike D20, D400 does not provide an image of the entire lesion and the selection of the areas to be imaged is operator-dependent. Interestingly, the videodermoscope that acquires the D400 images can progressively increase the magnification and therefore it can be useful to target an area of interest at D20 and to progressively zoom in. Moreover, there is no automatic focus and the operator needs to adjust the focus when acquiring the images. Another limitation of this study is the lack of correlation between histopathological images and images of other new non-invasive imaging techniques such as RCM. Moreover, our study did not include lentigo maligna and acral MM because of their peculiar features.

In conclusion, our study about the use of D400 for the diagnosis of lesions confirmed that D400 can reveal melanocytes’ irregular arrangement, shape and size in MMs, where they are found more frequently than in nevi; this can help the diagnosis of MM together with conventional dermoscopy data. Moreover, we could assume that D400 could help to direct the choice of the more representative site to perform a biopsy in case of large lesions and could help the identification of the anatomic structures observed under conventional ×20 dermoscopy.

## Figures and Tables

**Table 1:** Clinical features of the evaluated cases.

	Benign Lesions *n* = 117	Malignant Lesions *n* = 73	*p*-Value
Age (years; SD)	48.79 (19.19%)	64.98 (16.70%)	<0.001
Male	68 (61.3%)	31 (56.4%)	0.846
Female	49 (38.7%)	42 (43.6%)	

SD: standard deviation.

**Table 2:** ×20 dermoscopy features.

	Benign Lesions	Malignant Lesions	*p*-Value
7-point checklist			
Atypical pigment network	57 (48.7%)	59 (80.8%)	<0.001
Blue-whitish veil	18 (15.4%)	49 (67.1%)	<0.001
Atypical vascular pattern	1 (0.9%)	12 (16.4%)	<0.001
Irregular streaks	11 (9.4%)	22 (30.1%)	0.001
Regression structures	33 (28.2%)	49 (67.1%)	<0.001
Irregular pigmentations	40 (34.2%)	23 (31.5%)	0.823
Irregular dots/globules	36 (30.8%)	18 (24.7%)	0.457
General pattern			
Homogeneous	32 (27.4%)	43 (58.9%)	<0.001
Globular	41 (35.0%)	7 (9.6%)	<0.001
Network	67 (57.3%)	41 (56.2%)	1.000

**Table 3:** ×400 dermoscopy features.

	Benign Lesions	Malignant Lesions	*p*-Value
Cell presence	113 (96.6%)	72 (98.6%)	0.695
Keratinocytes	109 (93.2%)	64 (87.7%)	0.304
Roundish melanocytes	35 (29.9%)	36 (49.3%)	0.011
Dendritic melanocytes	19 (16.2%)	22 (30.1%)	0.037
Melanophages	19 (16.2%)	17 (23.3%)	0.310
Cell color			
Black	13 (11.1%)	7 (9.6%)	0.929
Brown	107 (91.5%)	66 (90.4%)	1.000
Violet/blue	45 (38.5%)	29 (39.7%)	0.983
Cell irregularity in shape and size	21 (17.9%)	38 (52.1%)	<0.001
Cell distribution: irregular arrangement	19 (16.2%)	30 (41.1%)	<0.001
Roundish nests	42 (35.9%)	22 (30.1%)	0.510
Out-of-focus structureless areas			
Bluish	36 (30.8%)	24 (32.9%)	0.886
Grey/brown	23 (19.7%)	11 (15.1%)	0.543
Vessels	26 (22.2%)	23 (31.5%)	0.210
Network			
with edged papillae	30 (25.6%)	3 (4.1%)	<0.001
without edged papillae	18 (15.4%)	19 (26.0%)	0.107
Angled nest	9 (7.7%)	16 (22.2%)	0.008

**Table 4:** Multivariate analysis.

	OR	*p*-Value
Age	1.05 (1.03–1.09)	<0.001
Irregular melanocyte distribution	4.69 (1.58–15.45)	0.007
Network with edged papillae	0.16 (0.03–0.57)	0.009

## Data Availability

Data is contained within the article and Supplementary Materials.

## Supplementary Materials

The following supporting information can be downloaded at: https://www.mdpi.com/article/10.3390/diagnostics13132238/s1.
