# Supplementary material for: Super-High Magnification Dermoscopy in 190 Clinically Atypical Pigmented Lesions
Source: Diagnostics (Basel). 2023 Jun 30;13(13):2238. doi: 10.3390/diagnostics13132238 (PMC10340569; doi:10.3390/diagnostics13132238)
Supplement: Supplementary file 1 [file diagnostics-13-02238-s001.zip › diagnostics-2315078-supplementary.pdf]

	Nevi				Melanoma			
	Trunk	Upper limb	Lower limb	pvalue	Trunk	Upper limb	Lower limb	pvalue
n	68	25	18		36	15	17	0.637
cells presence	67(98.5%)	23(92.0%)	17(94.4%)	0.290	35(97.2%)	15(100.0%)	17(100.0%)	0.975
Cell presence: Keratinocytes	66(97.1%)	22(88.0%)	15(83.3%)	0.077	32(88.9%)	13(86.7%)	15(88.2%)	0.070
Cell presence: Roundsh melanocytes	23(33.8%)	7(28.0%)	4(22.2%)	0.605	15(41.7%)	5(33.3%)	12(70.6%)	**0.002a,c**
Cell presence: Dendrititic melanocytes	11(16.2%)	5(20.0%)	3(16.7%)	0.909	9(25.0%)	10(66.7%)	2(11.8%)	0.299
Cell presence: Large polimorfous cellules violet (melanophages%)	13(19.1%)	4(16.0%)	2(11.1%)	0.715	7(19.4%)	6(40.0%)	4(23.5%)	0.345
Cell irregularity in shape and size (only for melanocytes%)	12(17.6%)	5(20.0%)	4(22.2%)	0.896	15(41.7%)	9(60.0%)	10(58.8%)	0.331
Cell color: black	8(11.8%)	2(8.0%)	2(11.1%)	0.873	2(5.6%)	1(6.7%)	3(17.6%)	0.869
Cell color: brown	65(95.6%)	21(84.0%)	16(88.9%)	0.169	33(91.7%)	14(93.3%)	15(88.2%)	0.246
Cell color: violet/blue	26(38.2%)	13(52.0%)	5(27.8%)	0.258	11(30.6%)	7(46.7%)	9(52.9%)	0.644
Cell distribution: irregular arrangement (only for melanocytes%)	11(16.2%)	6(24.0%)	2(11.1%)	0.513	12(33.3%)	7(46.7%)	7(41.2%)	0.403
Roundish nests (ex globules%)	24(35.3%)	9(36.0%)	8(44.4%)	0.770	8(22.2%)	6(40.0%)	4(23.5%)	0.564
out of focus bluish structureless areas	19(27.9%)	11(44.0%)	5(27.8%)	0.313	13(36.1%)	6(40.0%)	4(23.5%)	0.248
out of focus greay/brown structureless areas	12(17.6%)	2(8.0%)	7(38.9%)	**0.035c**	5(13.9%)	4(26.7%)	1(5.9%)	0.466
vessels	19(27.9%)	4(16.0%)	3(16.7%)	0.368	14(38.9%)	4(26.7%)	4(23.5%)	0.584
network with edged papillae	18(26.5%)	6(24.0%)	6(33.3%)	0.783	2(5.6%)	1(6.7%)	0(0.0%)	0.186
network without edged papillae	9(13.2%)	6(24.0%)	3(16.7%)	0.458	8(22.2%)	7(46.7%)	4(23.5%)	0.129
angulated nest	5(7.4%)	2(8.0%)	2(11.1%)	0.874	12(33.3%)	2(13.3%)	2(11.8%)	0.637
